# Pediatric sleep outcomes after endoscopy-directed simultaneous lingual tonsillectomy and epiglottopexy

**DOI:** 10.1186/s40463-022-00562-0

**Published:** 2022-03-14

**Authors:** Matthew Maksimoski, Sarah E. Maurrasse, Stephen R. Hoff, Jennifer Lavin, Taher Valika, Dana M. Thompson, Jonathan B. Ida

**Affiliations:** 1 Department of Otolaryngology – Head and Neck Surgery, Northwestern University Feinberg School of Medicine, Chicago, IL USA; 2 Division of Pediatric Otolaryngology – Head and Neck Surgery, Ann and Robert H. Lurie Children’s Hospital of Chicago, 225 E Chicago Ave, Chicago, IL 60611 USA; 3 Division of Otolaryngology—Head and Neck Surgery, Department of Surgery, Yale School of Medicine, New Haven, CT USA

**Keywords:** DISE, Airway surgery, Sleep disordered breathing, Obstructive sleep apnea, Airway obstruction, Evidence-based medicine

## Abstract

**Background:**

The purpose of this study was to evaluate the efficacy of sleep endoscopy-directed simultaneous lingual tonsillectomy and epiglottopexy in patients with sleep disordered breathing (SDB), including polysomnography (PSG) and swallowing outcomes.

**Methods:**

A retrospective review was performed of all patients undergoing simultaneous lingual tonsillectomy and epiglottopexy over the study period. PSG objective measures were recorded pre- and postoperatively, along with demographic data, comorbidities, and descriptive data of swallowing dysfunction in the postoperative setting.

**Results:**

A total of 24 patients met inclusion criteria for consideration, with 13 having valid pre- and postoperative PSG data. Successful surgery was achieved in 84.6% of patients, with no difference based on presence of medical comorbidities including Trisomy 21. Median reduction in obstructive apnea–hypopnea index (oAHI) with the procedure was 69.9%. Four patients (16.7%) had postoperative concern for dysphagia, but all objective swallowing evaluations were normal and no dietary modifications were necessary.

**Conclusion:**

Combination lingual tonsillectomy and epiglottopexy in indicated patients has a high rate of success in this single-institutional study without new dysphagia in this population. These procedures are amenable to a combination surgery in appropriately selected patients determined by sleep state endoscopy in the setting of SDB evaluated with drug-induced sleep endoscopy.

**Graphical abstract:**

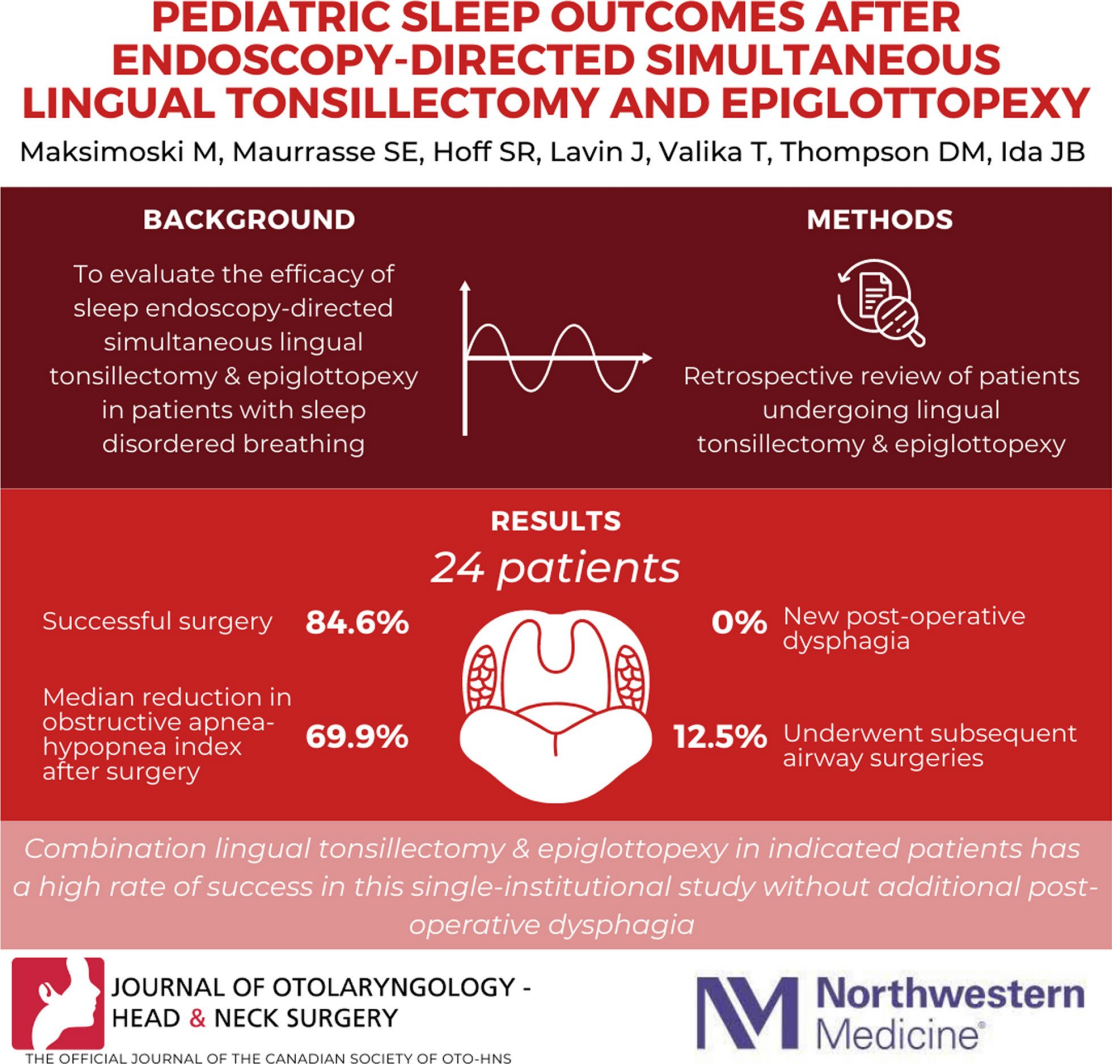

## Background

Pediatric sleep disordered breathing (SDB) encompasses a continuum of diagnoses from primary snoring to severe obstructive sleep apnea (OSA) [1–3]. First line treatment for uncomplicated pediatric patients with evidence of adenotonsillar hypertrophy and SDB is adenotonsillectomy as it leads to disease resolution in > 80% of patients without comorbidities [2, 4]. If adenotonsillectomy alone is not curative, or if patients have other risks such as Trisomy 21 [5], other anatomical sites may be contributing to obstruction [2, 3]. In patients with persistent symptoms after primary surgical intervention PSG is utilized to quantify obstruction and better direct consideration for non-invasive supportive medical management or additional surgical evaluation. Drug-induced sleep endoscopy (DISE) is a useful adjunct in identifying additional levels of obstruction which may be amenable to tailored surgical intervention [5, 6].

The base of tongue is the most common level of obstruction in children with persistent OSA after adenotonsillectomy [5–9]. This obstruction can be due to glossoptosis, lingual tonsillar hypertrophy, epiglottic retroflexion, or a combination of these. Children with medical co-morbidities, neuromuscular disorders and syndromic conditions such as Trisomy 21 are at higher risk of refractory OSA due to anatomic obstruction at the tongue base level, and may benefit from tailored surgical intervention [5, 10]. Lingual tonsillectomy has been shown to be effective for refractory OSA due to lingual tonsillar hypertrophy with a mean reduction in AHI of 4.7 to 8.9 in various studies [5–9, 11]. In addition, epiglottopexy has been described to address epiglottic retroflexion leading to obstruction. While simultaneous lingual tonsillectomy and epiglottopexy has been described [12], no study to date reports the safety and efficacy of this combination procedure. While removal of the lingual tonsil tissue and repositioning of the epiglottis can improve airway and airflow, rearrangement of this tissue could result in dysphagia. This study sought primarily to determine the effects of this combined procedure on objective measures of OSA outcomes. Secondarily, it sought to examine all patients undergoing this combination procedure for adverse swallowing outcomes, a concern of any aerodigestive tract surgery involving the epiglottolaryngeal complex.

## Materials and methods

Institutional review board approval was obtained for this study from the Ann and Robert H. Lurie Children’s Hospital of Chicago Institutional Review Board. A retrospective chart review was conducted to identify patients who underwent lingual tonsillectomy with simultaneous epiglottopexy between January 1, 2012 and December 31, 2020. Demographic data such as age, gender, body mass index (BMI), and medical and surgical history, including comorbidities, were extracted from the electronic medical record. Surgical details—including surgical technique—were also recorded. When applicable, sleep study data was collected including obstructive apnea–hypopnea index (oAHI), oAHI during rapid eye movement (REM) sleep, oxygen saturation nadir (OSN), maximum transcutaneous CO^2^ (CO^2^M), total sleep time spent below 89% oxygen saturation (TSTO), and percentage of sleep time spent with transcutaneous CO^2^ > 50 mmHg (TSTCO^2^). Patients were excluded from sleep result analysis if a pre- or post-operative PSG was absent. All sleep studies were performed in a dedicated pediatric sleep lab and interpreted by board certified sleep medicine physicians. Surgery was deemed successful if pre- and post-procedure data demonstrated a reduction in oAHI of 50% or a final oAHI of < 5 events per hour [7–9].

To determine whether swallowing function was altered by surgery, clinical notes and instrumental swallow evaluations were qualitatively reviewed. Data points included the presence or absence of clinical symptoms of dysphagia and when available, the results of instrumental evaluations including flexible endoscopic evaluations of swallowing (FEES) or videofluoroscopic swallow studies (VFSS). Pre- and post-operative screening in clinic was performed via detailed history with specific questioning of the patient and/or guardian for symptoms of dysphagia, including aspiration, coughing, delayed passage of food, aspiration pneumonia history, and results from previous instrumental evaluations of swallowing function.

Statistical analysis was conducted with SPSS statistical software (IBM-Armonk, NY). Statistical analyses include descriptive statistics for baseline characteristics and comorbidity data, paired T-tests for pre- and postoperative PSG quantitative data, and univariate analysis with Fisher’s exact test for subgroup analyses.

## Results

Twenty-four patients underwent simultaneous lingual tonsillectomy and epiglottopexy over the study period for lingual tonsillar hypertrophy and epiglottic prolapse seen on DISE in patients with SDB symptoms and/or PSG evidence of OSA. Of these patients, 20 (83.3%) underwent previous adenotonsillectomy while 4 (16.7%) had no evidence of adenotonsillar hypertrophy on examination and diagnoses of Trisomy 21, thus creating clinical concern high enough for performance of the DISE prior to an adenotonsillectomy. Patients were 62.5% male (15/24) and 37.5% female (9/24) and median age at surgery was 9.0 years with an interquartile range (IQR) of 4.0–14.0 years. Percentile medians for BMI, height, and weight were 71.02. (IQR: 41.09–89.59), 26.51 (IQR: 2.89–64.64), and 57.59 (IQR: 9.22–87.51) respectively. Comorbidities were common in this population; only one patient (4.5%) had OSA alone without another significant disease process at the time of surgery. Trisomy 21 occurred at a rate of 50.0% (12/24), and one patient was noted to have Trisomy 18. There were no differences between comorbidities (p = 1.00) or rates of chromosomal abnormalities (p = 0.347) when patients were stratified by preoperative OSA severity. Full data are listed below in Table 1.

**Table 1:** Demographics and characteristics of all patients undergoing simultaneous lingual tonsillectomy and epiglottopexy within the study population

Demographic characteristic	Frequency (%)	Median (IQR)
Total patients	24 (100%)	n/a
Male	15 (62.5%)	n/a
Medical comorbidity	23 (95.5%)	n/a
Trisomy 21	12 (50.0%)	n/a
Previous adenotonsillectomy	20 (83.3%)	n/a
Age	n/a	9.0 (4.0–14.0) years
BMI	n/a	71.1 (41.1–89.6) percentile
Height	n/a	26.5 (2.9–64.6) percentile
Weight	n/a	57.6 (9.2–87.5) percentile

IQR: interquartile range

Surgeries were performed by 5 different pediatric airway surgeons, ranging from 1 to 9 procedures per surgeon. Radiofrequency ablation (“Coblation”) was used in 75.0% (18/24) of the lingual tonsillectomies and a microdebrider was used in 20.8% (5/24). In one case, a carbon dioxide laser was used to remove lingual tonsil tissue. Coblation was used during the epiglottopexy step of all procedures to ablate the mucosa of the lingual surface of the epiglottis and foreshorten the glossoepiglottic ligament prior to suturing. Patients were followed for at least 12 months afterwards. Due to the tertiary nature of the primary clinic, many patients continued to follow up with their primary physician after this time period.

Thirteen patients had valid pre- and postoperative polysomnograms and were included in sleep results analysis (Table 2). Median preoperative oAHI was 11.0 (IQR: 9.3–20.2) with a median REM AHI of 32.35 (IQR: 12.2–48.5). The severity of pre-operative OSA varied: 8% of the cohort had mild OSA, 27% moderate, and 69% severe. Median postoperative oAHI was 2.0 (IQR 1.3–8.4) with a REM AHI of 7.6 (IQR: 3.0–19.7). Postoperative disease severity was as follows: 8% had no OSA, 46% had mild, 23% had moderate, and 23% had severe. Reduction in oAHI was noted in 92% of patients, with a median relative oAHI reduction of 69.9% (IQR: 53.6–83.8%, p < 0.05). The mean reduction of oAHI was 58.8% (95% CI 39.1%, 78.5%), including one patient who had mild OSA and a 25% increase in oAHI from 1.6 to 2.0. Windsorizing these data narrows the 95% CI to 50.4–81.2% with a mean reduction of 65.8% (p < 0.02).

**Table 2:** Characteristics of Patients undergoing simultaneous lingual tonsillectomy and epiglottopexy with pre- and post- operative polysomnography

ID#	Age	Sex	BMI percentile^A^	OSA diagnosis	Developmental comorbidity	Other comorbidity
1	14	Male	41.8	Severe	None	Epilepsy
2	15	Male	> 99.9	Severe	Trisomy 21	Steatohepatitis, asthma
3	14	Male	99.2	Moderate	None	Obesity, diabetes
4	3	Male	4.4	Severe	Trisomy 21	None
5	3	Female	98.7	Severe	None	Obesity
6	4	Female	93.8	Moderate	None	None
7	2	Male	56.6	Severe	Trisomy 21	Dysphagia
8	14	Male	77.0	Moderate	Trisomy 21	Asthma, dysphagia
9	14	Male	88.7	Severe	Trisomy 21	Hypothyroidism
10	12	Male	0.1	Mild	DPD, Cerebral palsy	Dysphagia, epilepsy
11	10	Male	16.9	Moderate	Trisomy 21	Hypothyroidism
12	9	Female	90.5	Severe	Trisomy 21	Hypothyroidism
13	14	Male	65.0	Severe	Trisomy 21	Hypothyroidism

*BMI* Body Mass Index, *OSA* Obstructive Sleep Apnea, *DPD* Dihydropyridine dehydrogenase deficiency

Median preoperative OSN was 84.5% (IQR: 79.0–89.0%), CO^2^M median was 52.0 (IQR: 48.0–54.0), TSTO median was 1.0 (IQR: 0.0–3.5), and TSTCO^2^ median was 0.5 (IQR: 0.0–2.1). Postoperative median OSN was 88.0% (IQR: 84.0–91.0%), CO^2^M was 52.0 (IQR: 49.0–53.0), TSTO was 0.6 (IQR: 0.0–0.8), and TSTCO^2^ was 0.0 (IQR: 0.0–1.5). Improvement in OSN was noted in 53.9% of patients, improvement in CO^2^M was noted in 33.3%, TSTO improved in 41.7%, and TSTCO^2^ improved in 33.3%.

A total of 77% of patients had a reduction in oAHI of > 50%, 54% had a final oAHI < 5 events per hour, and 69% noted a reduction in OSA diagnosis. In sum, surgery was successful in 84.6% of patients in this population. Subgroup analysis showed a greater likelihood of success (p = 0.011) in patients with a pre-operative diagnosis of moderate sleep apnea as compared to severe. Further analysis and PSG results are noted in Table 3. There was no difference in surgical success rate based on the presence of medical comorbidities of any kind (p = 1.00), diagnosis of Trisomy 21 (p = 1.00), or surgical technique (p = 1.00).

**Table 3:** Polysomnography results before and after lingual tonsillectomy and epiglottopexy

ID#	Preop oAHI	Preop O2 Nadir (%)	Preop OSA diagnosis	Mechanism of removal	Postop oAHI	Postop O2 Nadir	Postop OSA diagnosis	oAHI reduction (%)
1	10.9	89	Severe	Coblator	1.2	93	Mild	− 89.0
2	27.9	76	Severe	Coblator	26.2	73	Severe	− 6.1
3	9.3	94	Moderate	Coblator	0.7	95	None	− 92.5
4	79.7	71	Severe	Coblator	24.0	91	Severe	− 69.9
5	24.1	83	Severe	Coblator	18.0	76	Severe	− 25.3
6	8.0	86	Moderate	Microdebrider	1.3	84	Mild	− 83.8
7	10.2	79	Severe	Microdebrider	2.0	87	Mild	− 80.4
8	6.6	88	Moderate	Coblator	1.3	91	Mild	− 80.3
9	20.2	87	Severe	Coblator	8.4	84	Moderate	− 58.4
10	1.6	90	Mild	Coblator	2.0	87	Mild	+ 25.0
11	11.7	68	Severe	Coblator	1.1	95	Mild	− 91.6
12	11.0	90	Severe	Coblator	5.1	84	Moderate	− 53.6
13	14.2	80	Severe	Coblator	5.9	88	Moderate	− 58.5

*oAHI* Obstructive Apnea–Hypopnea Index, *OSA* Obstructive Sleep Apnea

All 24 patients were screened for dysphagia preoperatively with 5 patients (20.8%) requiring supplemental nutrition via a gastrostomy tube. All patients without preoperative signs of swallowing dysfunction were postoperatively screened for signs and symptoms of dysphagia. Patients with a positive screen (4/19, 21.1%) underwent further instrumental evaluation with FEES or VFSS. In all 4 patients, instrumental swallow evaluation results were normal. Screening for the 5 patients with preoperative dysphagia did not elucidate any worsening of their dysphagia, and one patient was able to transition to oral feeding following the procedure, eventually having a G tube removed. Therefore, the event rate for new postoperative swallowing anomalies was 0%. There were no postoperative bleeding events. However, 3 patients (12.5%) underwent subsequent airway surgeries to relieve persistent obstruction including one revision adenoidectomy and two tongue base suspensions.

## Discussion

Previous studies have examined the efficacy of lingual tonsillectomy alone, but none have analyzed the outcomes of simultaneous lingual tonsillectomy and epiglottopexy. In addition, there are no published data related to epiglottopexy as a standalone procedure, as retrodisplacement of the epiglottis may be secondary to tongue base anatomy. Given these limitations, it is difficult for pediatric otolaryngologists to appropriately counsel patients regarding the likelihood of success with sleep surgery directed at the tongue-base/epiglottic complex.

**Fig. 1:**
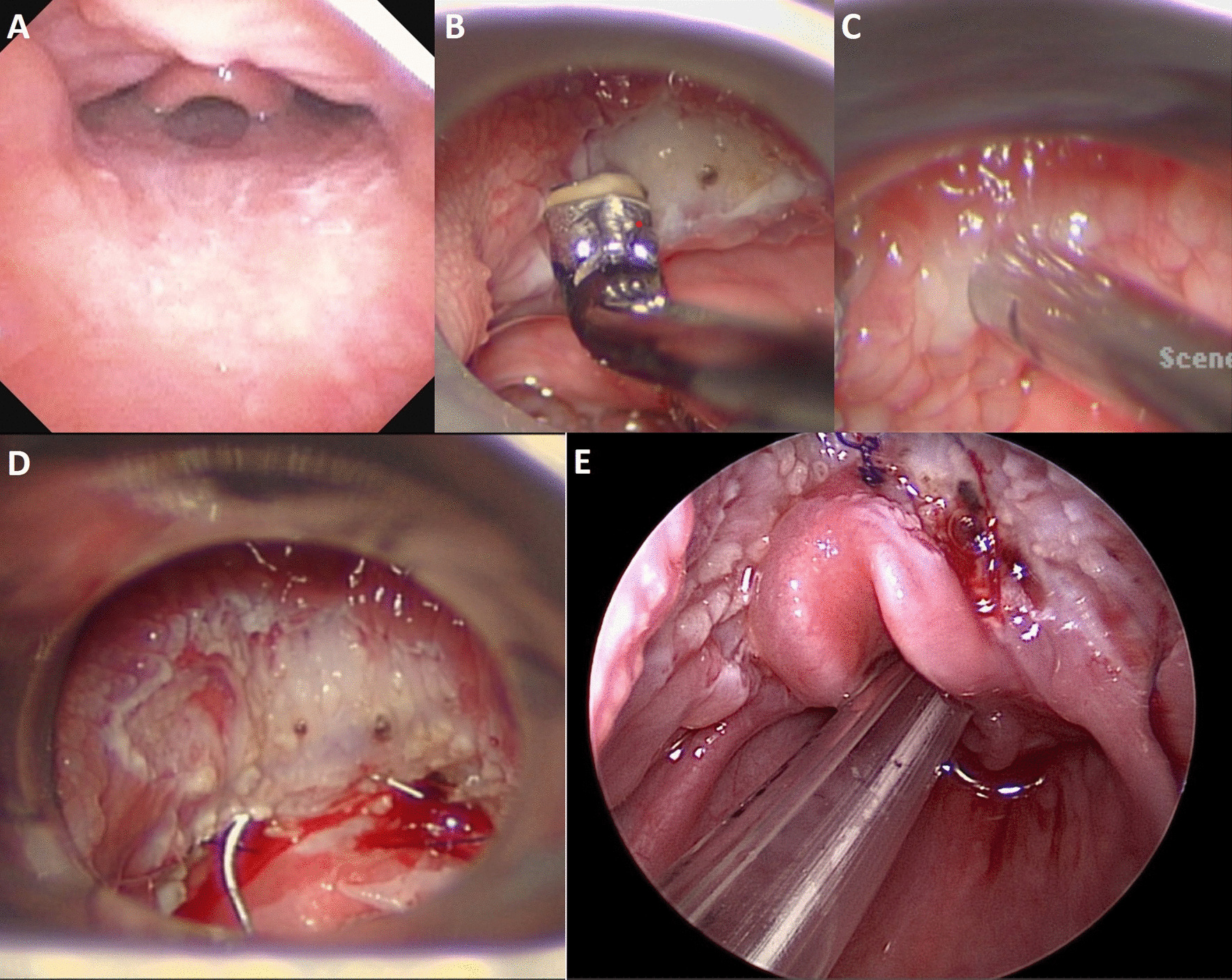
Photodocumentation of lingual tonsillectomy and epiglottopexy operative technique. **a** Preoperative lingual tonsillar hypertrophy and epiglottic prolapse, **b** Removal of lingual tonsil tissue with coblation, **c** Removal of lingual tonsil tissue with microdebrider, **d** Endoscopic suturing of the base of tongue to the lingual surface of the epiglottis, **e** Postoperative repositioning of the lingual tonsil-epiglottis complex with a widely patent airway

Lingual tonsillectomy is one of many surgical options for the treatment of OSA in the pediatric population, specifically for patients with hypertrophy of this tissue. Lingual tonsillectomy may be performed with any number of ablative techniques, such as monopolar suction cautery, laser, radiofrequency ablation (“Coblation”), or microdebridement. The lingual tonsil tissue can also be removed en bloc through the use of monopolar cautery, bipolar cautery, laser, robotic cautery, or cold steel [5–9, 12]. Epiglottopexy techniques vary; however, most agree that the procedure should involve some degree of mucosal disruption through superficial trauma or mucosal removal, followed by fixation of the epiglottis to the base of tongue (Fig. 1).

**Fig. 2:**
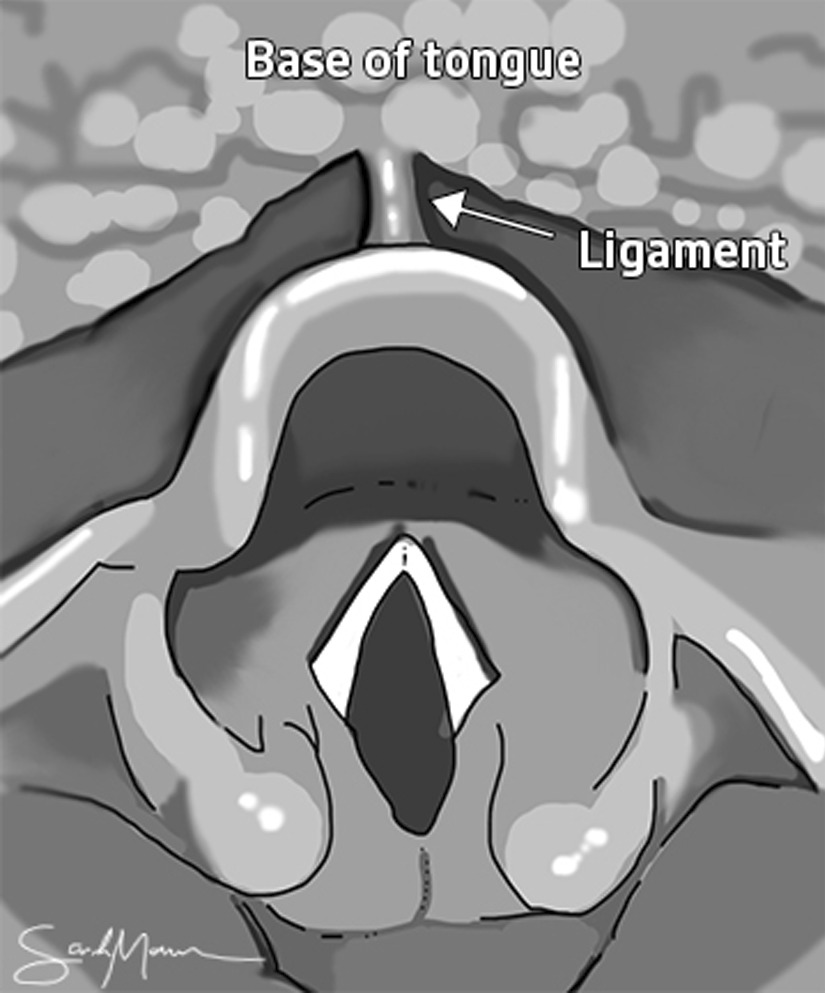
The relationship of the hyoepiglottic—or lingual-epiglottic—ligament (arrow) to the base of tongue makes it an important suspensory support for the epiglottis

Variability exists in the role of aryepiglottic fold trimming, mechanism of suture fixation, suture selection, suture placement, and treatment of the hyoepiglottic, or lingual-epiglottic, ligament [12–15]. Within the study institution, a non-absorbable suture is placed in a horizontal mattress format between the lingual surface of the epiglottis and the base of tongue. The hypoepiglottic ligament is foreshortened by cauterization with Coblation prior to suture placement. The hyoepiglottic ligament plays an important role in suspending the epiglottis to the base of tongue (Fig. 2). Depending on the technique used, lingual tonsillectomy can disrupt its structural integrity, leading to epiglottic retroflexion and secondary obstruction. Many surgeons anecdotally feel that epiglottopexy prevents this collapse by recreating the tension vector of the ligament and improving epiglottic stability, however studies regarding this have not been published.

In this cohort, concurrent lingual tonsillectomy and epiglottopexy led to a statistically significant reduction in oAHI (p < 0.05) and a reduction in severity of obstructive sleep apnea (p < 0.01). Additional subgroup analysis showed that patients with a preoperative diagnosis of moderate OSA had a significantly (p = 0.011) greater relative reduction in oAHI compared to the severe group. The most likely reason for this is that patients with severe sleep apnea often have significant multilevel obstruction, even if the base of tongue is the most notable. Additionally, as severe sleep apnea has no upper limit, even a large reduction in oAHI can result in a final number that is considered severe. Conversely, a similar absolute oAHI decrease in a patient with moderate OSA could downgrade the patient to the mild category.

The success rate in this cohort was 84.6%, which is higher than the range reported in studies of lingual tonsillectomy alone (51–62%) [5–9]. Additionally, of the 9 patients who did not have postoperative PSGs performed, 5 did not due to significant clinical improvement, while 2/5 patients without preoperative PSGs had postoperative tests showing oAHI < 5 events/hour. Rather than suggesting epiglottopexy should be performed on all patients undergoing lingual tonsillectomy, these results demonstrate concurrent surgery can be safe and effective when indicated based on DISE, congruent with previous publications on lingual tonsillectomy. These results further support the role of DISE-directed pediatric airway surgery in the management of refractory or complex airway obstruction.

The included cohort includes a large percentage of patients who have Trisomy 21 or Trisomy 18, making up 54.2% of patients. Although this is much higher than a representative portion of the general pediatric population, the risk of multilevel airway obstruction and base of tongue obstruction in patients with Trisomy 21 has been well-established [2, 4, 6, 8, 9]. Although this does limit generalizability of the data to all pediatric patients, the authors do not believe this rate of patients with chromosomal abnormalities significantly differs from the patient population of children with dynamic airway collapse on DISE which is amenable to repair with lingual tonsillectomy and epiglottopexy. Within our institution, a PSG is preformed when patients have symptoms of SDB without adenotonsillar hypertrophy; and if OSA is present a DISE is performed. Additionally, patients who have persistent OSA after an adenotonsillectomy undergo a DISE.

One patient did note an increase rather than a decrease in AHI from 1.6 to 2.0 events per hour. This increase in AHI of 25%, however, represents a variance of 1 breath every 2.5 h, leading the authors to believe this may be an insignificant change. Additionally, this particular patient noted a decrease in REM oAHI of 73.2%, from 11.2 to 3.0. Night-to-night variability in PSG within adult patients with OSA is well-documented [16–18], although data specifically geared towards pediatric patients are somewhat varied in their conclusions [19–23]. Based on levels of success from other patients and a small absolute change in oAHI, this particular patient’s results were believed to be due to PSG variability rather than a worsening of sleep parameters in the postoperative setting, especially given the dissonance between oAHI and REM oAHI.

Two patients with valid pre- and postoperative PSG data did not meet criteria for successful surgery (Patients 2 and 5 in the below tables). Other than severe obstructive sleep apnea, these patients had little in common precluding definitive conclusions as to possible risks of unsuccessful surgery. Patient 2 had many risk factors for OSA not amenable to surgical treatment and generalized hypotonia including trisomy 21, hypothyroidism, non-alcoholic steatohepatitis, severe asthma, and obesity. Patient 5 had cerebral palsy with hypotonia, significant developmental delay, seizures, scoliosis, and dihydropyrimidine dehydrogenase deficiency. Although both of these patients do have hypotonia, there were many patients within the cohort with similar risk factors (> 50% with trisomy 21 within this population) who reached surgical success. Interestingly, these two patients were entirely separate from the three patients who underwent subsequent sleep surgeries. Two of these patients did not have a preoperative PSG, while the other achieved a reduction in oAHI of 55.7 events per hour (69.9%) from 79.7 to 24.0.

There were no incidences of bleeding or dysphagia identified with this simultaneous procedure in the examined cohort. Only one previous study—a series of five patients—has addressed this, finding no increased incidence of postoperative dysphagia, consistent with the results therein. [13].

With relatively uncommon procedures, such as the one described here, some differences in surgical technique and instrumentation invariably exist [5–9, 12]. Previously described with the use of coblation and laser, within this cohort three surgeons performed the procedure entirely with coblation and the fourth with microdebrider and coblation. Given the relatively small cohort and multiple other confounders, it is difficult to draw any conclusions regarding the optimal surgical method. However, analysis showed no statistical difference in sleep outcomes when comparing the two methods. Larger studies with more surgeons would be needed to sufficiently power an analysis of optimal technique.

Major limitations of this study are inherent to its retrospective nature and the relative rarity of this procedure leading to a small patient cohort. Surgeries were performed by a variety of attending physicians and trainees, leading to variability in the technical steps of the surgery. Additionally, only a bit more than half of the patients (13/24) underwent both a pre- and postoperative sleep study. Although this was due to many factors, such as resolution of clinical symptoms or transition of care outside of the hospital system, it draws attention to the need for standardized care in these complex patients, which can drive opportunities for clinical research. These patients were included in general information on the demographics, and indications and complications of surgery to lessen risk of a type 2 error, but excluded from objective sleep analysis for completeness. There is a large component of patients within the study population with chromosomal abnormalities, which limits applicability to all pediatric patients. Additionally, while multiple surgeons performed the procedures, they were all performed at one institution, which may limit generalizability in separate practice environments. Patients were followed for less than 5 years, which does introduce the possibility of long-term sequelae from the surgery, or a lack of preservation of results.

As a relatively large cohort for an uncommon procedure, this study represents a significant contribution to the existing literature on tongue base surgery for refractory pediatric OSA. This study provides further data for targeted upper airway intervention for OSA, contextualized with previously existing literature documenting success of lingual tonsillectomy as a standalone procedure. While algorithms in management have been established previously, this expands on those to include information on steps that can be taken after levels of obstruction are identified to successfully treat OSA surgically in children [24]. Certainly, more research is required to better elucidate optimal candidacy for and clinical results of DISE-directed sleep surgery. However, for this cohort of patients—with lingual tonsillar hypertrophy and epiglottic prolapse—simultaneous correction of these levels was both safe and effective. This provides further data to support targeted upper airway intervention for complex OSA.

## Conclusion

Patient selection and preoperative work up—including drug induced sleep endoscopy—remain paramount for the appropriate management of residual OSA after adenotonsillectomy, in patients without adenotonsillar hypertrophy, or in those with Trisomy 21. When indicated, concurrent lingual tonsillectomy and epiglottopexy for lingual tonsillar hypertrophy and epligottic retroflexion is safe and effective in the pediatric population. This combination procedure can have a high surgical success rate, even in patients with significant comorbidities. Based on swallowing outcomes in this cohort, there is no evidence of increased risk for dysphagia following the combination procedure.

## Data Availability

All data generated or analyzed during this study are included in this published article.

## Abbreviations

SDB: Sleep disordered breathing
OSA: Obstructive sleep apnea
DISE: Drug-induced sleep endoscopy
PSG: Polysomnogram
BMI: Body mass index
oAHI: Obstructive apnea hypopnea index
REM: Rapid eye movement
OSN: Oxygen saturation nadir
CO^2^M: Maximum carbon dioxide saturation
TSTO: Total sleep time spent with oxygen saturation less than 89%
TSTCO^2^: Total sleep time spent with carbon dioxide greater than > 50 mmHg
FEES: Fiberoptic endoscopic evaluation of swallowing
VFSS: Video fluoroscopic swallow study
IQR: Interquartile range
CI: Confidence interval

## References

[1] Guilleminault C, Lee JH, Chan A (2005). Pediatric obstructive sleep apnea syndrome. Arch Pediatr Adolesc Med.

[2] Mitchell RB, Archer SM, Ishman SL, Rosenfeld RM (2019). Clinical practice guideline: tonsillectomy in children (update). Otolaryngol Head Neck Surg.

[3] Benedek P, Balakrishnan K, Cunningham MJ, Boudewyns A (2020). International Pediatric Otolaryngology group (IPOG) consensus on the diagnosis and management of pediatric obstructive sleep apnea (OSA). Int J Pediatr Otorhinolaryngol.

[4] Kang KT, Koltai PJ, Lee CH, Lin MT, Hsu WC (2017). Lingual tonsillectomy for treatment of pediatric obstructive sleep apnea: a meta-analysis. JAMA Otolaryngol Head Neck Surg.

[5] Kirkham E, Ma CC, Filipek N, Parikh SR (2020). Polysomnography outcomes of sleep endoscopy-directed intervention in surgically naïve children at risk for persistent obstructive sleep apnea. Sleep Breath.

[6] Xu Z, Wu Y, Tai J, Ni X, et. al. Risk factors of obstructive sleep apnea syndrome in children. J Otolaryngol Head Neck Surg. 2020; 49(11) 10.1186/s40463-020-0404-1 PMC7057627 32131901

[7] DeMarcantonio MA, Senser E, Meinzen-Derr J, Roetting N, Shott S, Ishman SL (2016). The safety and efficacy of pediatric lingual tonsillectomy. Int J Pediatr Otorhinolaryngol.

[8] Ishman SL, Chang KW, Kennedy AA (2018). Techniques for evaluation and management of tongue-base obstruction in pediatric obstructive sleep apnea. Curr Opin Otolaryngol Head Neck Surg.

[9] Prosser JD, Shott SR, Rodriguez O, Simakajornboon N, Meinzen-Derr J, Ishman SL (2017). Polysomnographic outcomes following lingual tonsillectomy for persistent obstructive sleep apnea in down syndrome. Laryngoscope.

[10] Cousineau J, Prévost AS, Battista MC, et al. Management of obstructive sleep apnea in children: a Canada-wide survey. J Otolaryngol Head Neck Surg. 2021; 50(53) 10.1186/s40463-021-00539-5 PMC8408936 34465374

[11] Rosen D (2011). Management of obstructive sleep apnea associated with Down syndrome and other craniofacial dysmorphologies. Curr Opin Pulm Med.

[12] Oomen KP, Modi VK (2014). Epiglottopexy with and without lingual tonsillectomy. Laryngoscope.

[13] Kanotra SP, Givens VB, Keith B (2020). Swallowing outcomes after pediatric epiglottopexy. Eur Arch Otorhinolaryngol.

[14] Schraff SADD (2005). Labioglossopexy and epiglottopexy. Oper Tech Otolaryngol Head Neck Surg.

[15] Whymark AD, Clement WA, Kubba H, Geddes NK (2006). Laser epiglottopexy for laryngomalacia: 10 years' experience in the west of Scotland. Arch Otolaryngol Head Neck Surg.

[16] Anitua E, Duran-Cantolla J, Almeida GZ, Alkhraisat MH (2019). Predicting the night-to-night variability in the severity of obstructive sleep apnea: the case of the standard error of measurement. Sleep Sci.

[17] Mjid M, Ouahchi Y, Toujani S, Beji M (2016). Night-to-night variability of the obstructive sleep apnoea-hypopnoea syndrome. Rev Mal Respir.

[18] Stöberl AS, Schwarz EI, Haile SR, Kohler M (2017). Night-to-night variability of obstructive sleep apnea. J Sleep Res.

[19] Katz ES, Greene MG, Carson KA, Marcus CL (2002). Night-to-night variability of polysomnography in children with suspected obstructive sleep apnea. J Pediatr.

[20] Orntoft M, Andersen IG, Homoe P (2020). Night-to-night variability in respiratory parameters in children and adolescents examined for obstructive sleep apnea. Int J Ped Otorhinolaryng.

[21] Eralp EE, Yegit CY, Gokdemir Y, Ersu R (2020). Night-to-night variability of polygraphy in children with obstructive sleep apnea. Eur Resp Journ.

[22] Kontos A, Baumert M, Lushington K, Martin J (2020). The inconsistent nature of heart rate variability during sleep in normal children and adolescents. Front Cardiovasc Med.

[23] X. L. R. Hoppenbrouwer et al. Night to night pulse oximetry variability in children with suspected sleep apnea. In: 2018 40th Annual International Conference of the IEEE Engineering in Medicine and Biology Society (EMBC). 2018; pp. 179–182 10.1109/EMBC.2018.8512216 30440367

[24] Heath DS, El-Hakin H, Al-Rahji Y, et al. Development of a pediatric obstructive sleep apnea triage algorithm. J Otolaryngol Head Neck Surg. 2021; 50(48) 10.1186/s40463-021-00528-8 PMC8281470 34266488

